# Can Blood Oxygenation Level Dependent Functional Magnetic Resonance Imaging Be Used Accurately to Compare Older and Younger Populations? A Mini Literature Review

**DOI:** 10.3389/fnagi.2018.00371

**Published:** 2018-11-13

**Authors:** Melissa E. Wright, Richard G. Wise

**Affiliations:** ^1^Cardiff University Brain Imaging Research Center, School of Psychology, Cardiff University, Cardiff, United Kingdom; ^2^School of Optometry and Vision Sciences, Cardiff University, Cardiff, United Kingdom

**Keywords:** aging, brain imaging, cerebral blood flow, cerebral hemodynamics, fMRI, neurovascular coupling

## Abstract

A wealth of research has investigated the aging brain using blood oxygenation level dependent functional MRI [Blood oxygen level dependent (BOLD) functional magnetic resonance imaging (fMRI)]. However, many studies do not consider the aging of the cerebrovascular system, which can influence the BOLD signal independently from neural activity, limiting what can be inferred when comparing age groups. Here, we discuss the ways in which the aging neurovascular system can impact BOLD fMRI, the consequences for age-group comparisons and possible strategies for mitigation. While BOLD fMRI is a valuable tool in this context, this review highlights the importance of consideration of vascular confounds.

## Introduction

With an expanding older population, research into the aging brain has become increasingly important. Blood oxygen level dependent (BOLD) an functional magnetic resonance imaging (fMRI) has often been applied to investigate how age affects neural function; common findings include increased task-induced activation in frontal areas (Park et al., 2003; Langenecker et al., 2004; Gutchess et al., 2005), decreased activation in occipital (Madden, 2004) and temporal areas (Park et al., 2003; Gutchess et al., 2005), and altered functional connectivity (Dørum et al., 2016) with increasing age.

**FIGURE 1:**
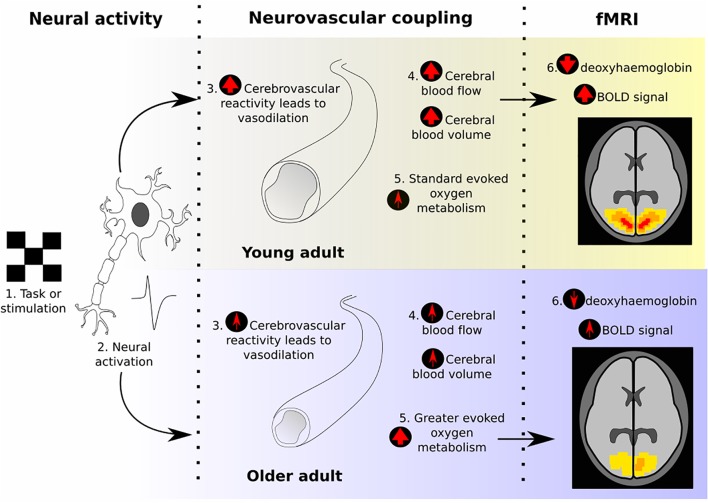
Illustration of the neurovascular coupling processes that give rise to the BOLD response, and how these typically change between young and old adults, according to previous literature. The final “fMRI” section demonstrates what effect would be expected on the BOLD response due to these age-related changes, assuming equal neural activity.

However, the BOLD signal does not directly index changes in neural activity (as summarized in Figure 1). The BOLD signal reflects the balance between changes in cerebral blood flow (CBF), determined by the characteristics of neurovascular coupling (NVC), and changes in tissue oxygen consumption. Following increased neural activation, the signaling processes that underlie NVC lead to vasodilation and increased blood flow, tending to increase BOLD signal. Simultaneously, the increased oxygen consumption, needed to fuel the increased neural activity, will decrease the BOLD signal. The picture is complicated further by the additional dependence of BOLD signal changes on the volume of the relevant venous blood compartment (cerebral blood volume, CBV) and the local vascular architecture.

This complexity leaves BOLD fMRI open to confounds when comparing age groups. If there are age-related differences in NVC or oxygen consumption, then a difference in BOLD signal would be produced independently of a difference in neural activation. Therefore, the interpretability of any BOLD fMRI studies that compare age groups without neurovascular controls must be questioned. Here, we review how aging impacts the neurovascular system and brain metabolism and how age may confound BOLD fMRI studies, as well as discuss strategies for addressing this issue.

## Aging Neurovascular System

### Vasculature

Firstly, the structural integrity of the cerebral vasculature is compromised during aging (Gazzaley and D’Esposito, 2004). This includes thickening of the capillary basement membrane (Alba et al., 2004) and decreases in capillary density, though the latter has more mixed evidence (Meiger-Ruge et al., 1980; Kalaria, 1996). While the differing results for capillary density may reflect different methodologies and variability in neurovascular health (especially as reported changes tend not to be large; Riddle et al., 2003), they could also be due to the age ranges included. Specifically, decreases in capillary density may only begin in later senescence; Brown et al. (2007) found a stronger negative correlation with age and density when only considering participants over 60 years old. Therefore, this trend may not be visible in younger age ranges. Structural changes (which impact vessel elasticity and functionality) would influence CBF and CBV dynamic changes, which then impact BOLD signal magnitude and timing (Brown et al., 2003). For example, it has been suggested that age-related arterial stiffness, which is especially seen centrally (O’Rourke and Hashimoto, 2007), may lead to CBF pulsatility increases despite overall CBF decreases (Tarumi et al., 2014). A loss of vessels is also especially important when comparing across aging studies, as different magnet strengths and pulse sequences bias the BOLD signal to different vessel sizes (Mueller-Bierl et al., 2007).

### Cerebral Blood Flow (CBF)

Lower resting-CBF has been demonstrated in older adults using arterial spin labelling (ASL) fMRI (Restom et al., 2007), even when potential confounds such as partial volume effects and age-related cortical thinning have been corrected for (Asllani et al., 2009; Chen et al., 2011). This has been replicated with different scanning techniques, though the magnitude differs across regions (Martin et al., 1991; Bertsch et al., 2009; Chen et al., 2011). It is suggested that this CBF decrease is due to age-related differences in CO_2_ end-tidal partial pressure, which reflects CO_2_ arterial tension, rather than an independent effect of age (De Vis et al., 2015). CBF is an important parameter in determining the BOLD response, with studies finding an inverse correlation between basal CBF and BOLD signal change, (Cohen et al., 2002; Brown et al., 2003; Stefanovic et al., 2006) in line with the deoxy-hemoglobin dilution model (Hoge et al., 1999). Indeed, age-related BOLD differences have been shown to significantly decrease when lower CBF is accounted for, suggesting a substantial vascular contribution to the observed BOLD age-differences (Zebrowitz et al., 2016).

### Cerebrovascular Reactivity (CVR)

Age also influences Cerebrovascular Reactivity (CVR), which refers to the increase in CBF and CBV following exposure to a vasodilatory stimulus, such as after neural activity in order to meet the increased energy demand. Neurovascular coupling relies on CVR being preserved. A decrease in vasodilatory capacity is reported in aged rats (Tamaki et al., 1995). Flück et al. (2014) used transcranial Doppler ultrasound (TCD) to investigate this in humans and concluded that older adults had reduced CVR, which may be due to vascular stiffening. However, in TCD the blood velocities through the posterior and middle cerebral arteries are measured rather than absolute CBF, so there may be unobserved regional dependences, and the lack of direct tissue measurement of CBF using TCD means that results should be interpreted with caution (Coverdale et al., 2014; Reinstrup et al., 2014). Other studies have used a CO_2_-inhalation task (Liu et al., 2013a) or breath-holding challenge, (Riecker et al., 2003) in which BOLD fMRI has been used monitor evoked-CBF changes. Despite breath-holding challenges having potential confounds (e.g., metabolic rate or lung function can influence the quantity of blood CO_2_) and not showing a linear relationship between holding time and arterial partial pressure of CO_2_, (Fierstra et al., 2013) similar results were found between breath-hold and CO_2_ inhalation. For both methods, older adults showed lower CVR. Importantly, while uncorrected BOLD fMRI in Liu et al. (2013a) implied decreases in task-related V1 and medial temporal lobe activity with age, this effect disappeared when CVR was corrected for. Additionally, a stronger increase in bilateral frontal gyrus activation in older adults was found after CVR-correction, which may reflect compensatory mechanisms as there was no difference in memory scores (Liu et al., 2013a). This provides strong evidence for an age-related decrease in CVR; this would lead to a smaller amount of vasodilation and subsequent evoked CBF, deoxyhaemoglobin, and BOLD signal, causing an under-representation of neural responses in older groups when comparing across ages.

### Cerebral Metabolic Rate of Oxygen (CMRO_2_)

Another key factor regulating BOLD signal is the rate at which oxygen is extracted from the blood and used for energy release (rate of cerebral metabolic oxygen consumption; CMRO_2_). An increase in CMRO_2_ would lead to an increase in deoxy-hemoglobin in the venous vessels, and thus a decrease in BOLD signal due to the paramagnetic properties of deoxy-hemoglobin (Schwarzbauer and Heinke, 1999). Although age-related changes in resting CMRO_2_ have been reported, there is conflicting evidence as to their direction. Peng et al. (2014) investigated this using CBF, blood oxygen saturation percentage, and total blood oxygen capacity to estimate resting CMRO_2_, which is suggested to be a reliable method (Liu et al., 2013b). They found a significant increase in CMRO_2_ in older adults, which could act as a compensatory mechanism for declining cognitive functions or weakening CVR and CBF. Conversely, De Vis et al. (2015) reported a decrease in baseline CMRO_2_, which was present in all regions except for the occipital cortex (although the parietal cortex lost significance once end-tidal partial pressure of CO_2_ was controlled for). This conflicting result is reflected in other studies, using similar and alternative techniques (Lu et al., 2011; Aanerud et al., 2012). It is possible that the method by which CMRO_2_ was derived influenced results, as different methods were used (e.g., ASL vs. phase-contrast flow velocity). It may also simply reflect the greater within- and between-subject variability that has been reported in older participants’ BOLD signals (Kannurpatti et al., 2010; Garrett et al., 2013; Baum and Beauchamp, 2014 but see). Of particular relevance for interpreting task-related BOLD signal changes is the difference in evoked CMRO_2_, of which several studies report an age-related increase (Restom et al., 2007; Hutchison et al., 2013). In the presence of an equal blood flow response across age groups this would have the effect of reducing the BOLD signal response.

Interactions between changes in NVC and oxygen consumption may have the greatest confounding effect; for example, alterations in CMRO_2_ and CBF may explain lower BOLD responses in older adults, as higher demand but diminished supply would lead to higher venous deoxy-hemoglobin concentrations (Lu et al., 2011; Hutchison et al., 2013). Furthermore, these effects are clinically silent and thus not excluded by controlling for health status (e.g., cardiac or neurological illness). Several studies have linked age-related changes in BOLD signal to neurovascular factors, such as signal timing (Taoka et al., 1998) and increased voxel-wise noise (D’Esposito et al., 1999). Another finding that confounds the comparison of age groups using BOLD fMRI is that of increased within- and between-subject variability that has been reported in older adults (e.g., Kannurpatti et al., 2010; Baum and Beauchamp, 2014). It may be that variations in NVC and levels of CMRO2 contribute to this greater variability (though this cannot be the only contributor, as increased neural variation is also found electro-physiologically; McIntosh et al., 2014). However, the extent of this increased variability with age was found to vary greatly based on the motion correction pipeline, despite the pipelines having similar goals, introducing extra difficulty in age group comparisons (Turner et al., 2015). Therefore, to better interpret age-related changes in BOLD signal, these factors must be considered and examined.

## Drug-Related Factors

Other age-related factors may also indirectly impact the neurovascular system. For example, older adults tend to use more prescription and over-the-counter drugs (Francis et al., 2005). Although studies often exclude certain medications, such as beta-blockers or anti-depressants, they rarely account for over-the-counter products such as non-steroidal anti-inflammatory drugs (NSAIDs). A survey of aspirin use (a common NSAID) in adults over 45 years old found that 52% reported current use, many did so for primary prevention, and that regular use was associated with markers of a healthy lifestyle (Williams et al., 2015). This indicates that over-the-counter NSAID use is higher in older adults, even if they are relatively healthy. If these medications alter NVC, it may confound the BOLD signal. There has been little research into this, though aspirin was found to reduce resting-CBF in an *in vivo* rabbit model, which was suggested to be due to inhibiting prostacyclin and/or nitric oxide (Bednar and Gross, 1999). A human study investigated the effects of two other NSAIDs, naproxen (available over-the-counter in lower doses) and indomethacin (prescription only). Using transcranial Doppler sonography, it found a significant decrease in resting and visually evoked blood velocity, possibly due to inhibiting vasodilatory processes (Szabo et al., 2014). These results may also be more reflective of average older adult NSAID use than other work as the medication was administered orally in usual doses over 2 days, rather than an injection (Jensen et al., 1993) or a single high dose (Markus et al., 1994). However, they used young adults so it is inconclusive whether the same effect would be evoked in the aged neurovascular system. Other types of NSAIDs may also have differing effects; for example, CBV and CBF didn’t change after short-term exposure to ibuprofen in piglets (Pellicer et al., 1999). While under-researched in older adults, increased use of NSAIDs may contribute to an inaccurate estimation of neural activity differences in older adults. Further studies should examine the influence of over-the-counter medications in older adults and on the BOLD response.

## Potential Controls

As non-neuronal factors independently alter the BOLD response, such factors should be understood and controlled for as far as possible to maximize the interpretability of fMRI data. One approach involves normalizing BOLD fMRI data with measure of the effectiveness of NVC, CVR being commonly used under the hypothesis that it explains a lot of variability in NVC. CVR can be estimated by altering arterial blood CO_2_ (a vasodilator), and measuring the CBF/BOLD increase. One approach involves a CO_2_-inhalation task with simultaneous fMRI recording and using the change in BOLD to normalize the task-related response (see Liu et al., 2013a). However, an inhalation task may prove strenuous for older participants. Breath-holding paradigms, which involve the participant naturally increasing blood CO_2_ by holding their breath, may be more tolerable and have shown comparable results (Kastrup et al., 2001; Handwerker et al., 2007). Breath-holding is suggested to be reliable even in those with poor breath-hold performance, such as older adults (Bright and Murphy, 2013). However, breath-holding can cause severe motion artifacts, particularly in high-field strengths, such as 7T. Confounds could also be caused by patient anxiety over a breath-hold or inhalation task, such as increased movement or increased heart rate, which may occur if an older participant finds the task difficult to perform.

Another possibility is resting state fluctuation amplitude (RSFA), which examines the signal amplitude variation when the participant is ‘task free’, thus reducing confounds such as head movement which may be seen with a breath-holding or a hypercapnia challenge (e.g., Hall et al., 2011). The RSFA has been suggested as an index of vascular contributions to variability in the BOLD response as it will depend on local CVR and CBV. Tsvetanov et al. (2015) used RSFA to scale BOLD data and found that the magnitude of age-related differences significantly decreased. Furthermore, though RSFA has been criticized for confounding together neural and neurovascular properties (Lipp et al., 2015), these contributions have been separated using magnetoencephalography and neurovascular function measures (Tsvetanov et al., 2015). Only vascular factors mediated age-effects on RSFA, which suggests that RSFA is largely driven by neurovascular contributions (Wolpe et al., 2016). However, RSFA has also been reported to have poorer repeatability and model fit than breath holding tasks (Lipp et al., 2015). Although these methods of estimating CVR have major limitations when applied to an older population, they offer an empirical way of reducing non-neural variability.

An alternative to trying to ‘correct’ the BOLD response is to measure a process that is likely to more closely reflect levels of neural activity, such as CMRO_2_. By calibrating fMRI to quantitatively examine the amount of oxygen metabolism, reflective of oxidative energy release, we avoid the influence of factors such as resting-CBF with established age differences. Methods have been proposed to map CMRO_2_ reliably, such as using combined hyperoxia and hypercapnia (Wise et al., 2013; Germuska et al., 2016). However, this again involves gas inhalation, which may be too strenuous or uncomfortable for very old participants. A method involving diffuse optical tomography with BOLD and ASL fMRI may therefore be better as it does not involve gas inhalation but was found to be highly correlated with previous methods (Yücel et al., 2014). This also does not assume a constant CMRO_2_, which is not always found (Xu et al., 2011).

Another option is implementing a control task that is assumed to not show neural age-related changes (e.g., a simple motor task; D’Esposito et al., 1999). A difference between groups would therefore suggest a global NVC confound. The signal and noise characteristics (i.e., the haemodynamic response function) within each group could then be characterized to create a global normalization factor (Samanez-Larkin and D’Esposito, 2008), assuming that it can be extended across the brain. However, this approach assumes equivalent neural activity across age groups, which may not be valid even with simple motor tasks(Sailer et al., 2000; Inuggi et al., 2011). It also does not account for region-dependant variation in neurovascular function (Ances et al., 2008; Devonshire et al., 2012). Additionally, the neurovascular system has different influences on BOLD signal depending on the task (Kannurpatti et al., 2010), possibly because they utilize different functional and structural networks. Signals characterized in control tasks therefore may not be generalizable.

The above controls may be best when used in tandem with designs intended to reduce neurovascular confounds, which also have the advantage of being easier to implement. Investigating relative change within groups or varying the conditions parametrically may be useful as they provide an internal control (Gazzaley and D’Esposito, 2004; Ankudowich et al., 2016). However, these designs will still be confounded by resting levels of CBF influencing the magnitude of evoked BOLD signal (Cohen et al., 2002). Another possible method is event-related fMRI, which allows the separation of cognitive processes; if age-related differences are found in one stage of a cognitive task but not another, which both use similar brain areas (e.g., memory encoding vs. retrieval), observed differences are more likely to be neuronal in origin (Rypma and D’Esposito, 2000; Gazzaley and D’Esposito, 2004). Although the methodological design depends heavily on the research question, designs such as the above may be useful for comparing age groups with BOLD fMRI.

## Conclusion

The BOLD signal reflects the complex interaction of many factors, such as CVR, CBF, and CMRO_2_. When using BOLD fMRI to compare across age groups, researchers must be aware that age can impact the majority of these non-neural factors, so differences across age groups cannot be solely contributed to neuronal changes. Neuronal changes, if present, may be exaggerated or masked by age-related alterations in factors such as resting-CBF. This requires careful consideration during study design and interpretation, so as not to falsely highlight neuronal changes that are not present or miss out on identifying neuronal changes that are present.

Finally, it should be noted that most of the neurovascular changes noted are part of the normal aging process and may have an independent effect on cognition (Moorhouse and Rockwood, 2008; Bangen et al., 2014). For example, neurovascular uncoupling, without CBF changes, leads to significant cognitive impairment in mice (Tarantini et al., 2015). Monitoring these factors using fMRI also has interesting applications in clinically assessing neurovascular health. While BOLD fMRI requires careful consideration if interpreting signals as neuronal in origin, more broadly it demonstrates great potential as a tool, in aging research, that is sensitive to neurovascular and neurometabolic changes.

## Author Contributions

MW conceived the review, literature search, and wrote the manuscript. RW conceived the review and revised the manuscript.

## Conflict of Interest Statement

The authors declare that the research was conducted in the absence of any commercial or financial relationships that could be construed as a potential conflict of interest.
